# Umbilical cord care practices and associated factors among mothers who gave birth in the last six months in hetosa district, Arsi zone, Ethiopia 2021: Community-based mixed design

**DOI:** 10.1016/j.heliyon.2024.e41133

**Published:** 2024-12-13

**Authors:** Genat Balcha Abdi, Bekalu Kassie Alemu, Tensae KassaYizengaw, Beker Ahmed Hussein

**Affiliations:** a Arsi University, Department of Midwifery, Asella, Ethiopia; b Debre Markos University, Department of Midwifery, Debre Markos, Ethiopia

**Keywords:** Cord care practice, Qualitative, Associated factors, Arsi zone

## Abstract

**Background:**

Cord care is the series of steps applied to handle the umbilical cord after delivery of the newborn. Despite increasing the number of primary health facilities, unhygienic cord care remains persist.

**Objective:**

To assess umbilical cord care practices and associated factors among mothers who gave birth in the last six months in Hetosa district, Arsi zone, Ethiopia, 2021.

**Methods:**

A mixed-type cross-sectional study design was conducted in the Hetosa district of Arsi zone, from April 15 to May 15, 2021, with 550 mothers using a systematic random sampling technique for quantitative study. Quantitative Data were collected using a structured pretested questionnaire, entered into EpiData version 3.1, and then exported to SPSS version 25 for analysis. Factors associated with outcome variables were identified using logistic regression analysis. Qualitative data was collected through twelve in-depth interviews and analyzed thematically.

**Result:**

Overall good cord care practice was 53.4 % [95%CI, 49, 58]. Being in age group ≤ 24years (AOR = 4.55, 95 % CI; 2.08, 9.98) and 24–32years (AOR = 3.70, 95 % CI; 1.98, 6.94), among those who attend primary school (AOR = 5.26, 95 % CI: 2.50, 11.04) and secondary and above (AOR = 3.63, 95 % CI: 1.61, 8.18), health facility delivery (AOR = 5.09, 95 % CI; 2.95, 8.78), and having good knowledge about cord care(AOR = 8.58, 95 % CI; 5.09, 14.46) were statically significantly associated with good umbilical cord care practice. Participants believed that applying fresh butter around the stump could protect the baby.

**Conclusion:**

and recommendation: The study found that the percentage of mothers practicing good cord care was low. To improve this, policies and strategies should focus on women's education, encouraging institutional delivery and family planning use among aged mothers, and health education during ANC visits and addressing community beliefs through culturally sensitive health promotion strategies particularly focusing on older mothers and traditional birth attendants to improve cord care practices.

## 1 Introduction

The umbilical cord is a delicate, adaptable structure that associates the embryo to the mother through the placenta from the 6th week of pregnancy until birth. It contains one vein, which carries oxygenated, nutrient-rich blood to the fetus, and two arteries which carry deoxygenated, nutrient-depleted blood away from the fetus. The stump continuously dries, shrinks, and isolates from the body, typically somewhere in the range of 5 and 15 days after birth with shading change from a yellow-green to dark as it dries out. During this period, the umbilical cord ought to be kept perfect and dry to dodge contamination [1].

Care of the umbilical cord is the series of care applied in handling of the umbilical cord after delivery of the newborn. This includes clamping, cutting, and serial daily care of the umbilical stump post-delivery. These actions, if not meticulously carried out will contribute significantly to the newborn risk of neonatal infections and mortality [2]. Umbilical cord infections contribute significantly to neonatal deaths. In sub-Saharan Africa, neonatal infections are the most common cause of death among children under age five. Poor and unhygienic cord care practices are often to blame [3].

In countries with limited resources, umbilical cord infection continues to be a major cause of neonatal morbidity and poses a significant risk of mortality [4]. However, best practices for umbilical cord care (UC) remain controversial in developed countries with aseptic perinatal care and dry cord care [5].

Numerous further intercessions to forestall umbilical string contamination have been attempted. The two that have been studied most extensively are 70° alcohol and 4 % chlorhexidine, administered in different dosage forms (alcoholic or aqueous solution, gel or powder). Other antiseptics used for the purpose are triple dye (a combination of 3 disinfectant solutions frequently used in the United States), povidone, iodine, and salicylic acid, among others. Topical antibiotics such as silver sulfadiazine, tetracyclines, or neomycin have also been used. However, the application of any CHX to the umbilical cord of the newborn led to a 23 % reduction in all-cause neonatal mortality [4,6].

WHO recommends dry cord care for nations with good obstetric care and low neonatal mortality rates. Recent studies suggest applying chlorhexidine on the umbilical cord stump during the first week of life for neonates delivered at home in settings with high NMR of 30/1000 live births or higher [7]. The Ethiopian government introduced the Community Based Newborn Care (CBNC) program in 2013, to improve maternal and newborn health outcomes. Chlorhexidine was included as one of the components in CBNC after consensus building, and it was manufactured locally. So Chlorhexidine is a feasible and acceptable intervention in the Ethiopian setting [8,9]. In addition to this and antiseptic mothers' awareness about cord care practices and maternal higher educational level were important factors in maintaining the cord in a healthy state [10].

Cord infections in newborns range from 3 to 5% in developing countries and 0.5 % in developed ones. The WHO estimates over 4 million neonatal deaths occur each year, with 3.4 million in the first week, and the highest number of deaths within the first 24 h of life [11]. However, there are no recent studies regarding mothers' cord care practices including harmful traditional practices that might strongly contribute to newborn infection which leads to neonatal mortality and morbidity in Ethiopia as well as in the study area.

Neonatal mortality is high worldwide, especially in sub-Saharan Africa. Neonatal sepsis and tetanus are major causes of neonatal death, with neonatal sepsis responsible for over 15 % of neonatal deaths globally and is the third leading cause of death among infants in their first month of life [11,12].

. Some traditional substances, such as cow dung, herbal preparations, ash, mud, and oils, are often applied to promote healing and hasten cord separation [12]. However, the use of CHX in these situations may be considered to replace the application of a harmful traditional substance to the cord stump [13].

Bacterial colonization of the cord can result in omphalitis, thrombophlebitis, cellulitis, or necrotizing fasciitis. Various topical substances continue to be used for cord care around the world to mitigate the risk of serious infection [14].

Even though Chlorhexidine application delays the time to cord separation, its application to the cord reduces the risk of neonatal mortality and omphalitis in infants born at home in high-NMR settings [15]. A single intervention of 4 % CHX has been displayed by numerous studies to effectively reduce the incidence of omphalitis-related mortality by approximately 25 % [16].

The umbilical cord is a common entry point for bacteria causing neonatal sepsis. Harmful traditional practices related to umbilical cord care are influenced by cultural beliefs of mothers. However, there is limited information available on this topic in the study area and in Ethiopia. This study aims to fill this gap.

## 2 Methods & materials

### 2.1 Study design

A mixed quantitative community-based cross-sectional study, supplemented with a qualitative study design was conducted.

### 2.2 Study area and period

The study was conducted from April 15 to May 15, 2021, in Arsi Zone Hetosa district. The Hetosa District is one of the largest districts of Arsi Zone, Oromia Regional State of Central Ethiopia. It is located 160 Km from Addis Ababa, the capital city of Ethiopia. The district is typical of the zone in terms of population density, socio–cultural and economic state, and demographic conditions. It has a total of 25 (twenty-two rural and three urban) kebeles in number. The area lies between 08° 08′N-08°13′ E latitude and 39° 14′ N −39° 23′ E longitude with an elevation range from 1500-4170 m above sea level with an estimated total population of 178,229 and 36,597 households according to the 2007 national census which was conducted by the Central Statistical Agency of Ethiopia (CSA). The estimated total number of women of reproductive age group and women who gave birth in the last six months at the time of data collection in the district (both rural and urban) was 39,210 and 2248, respectively.

### 2.3 Population

#### 2.3.1 Source population

All mothers who gave birth in the last six months at the time of data collection in the Hetosa district were considered as a source population for the quantitative part of this study population.

#### 2.3.2 The study population for quantitative data

All mothers who gave birth in the last six months at the time of data collection in selected kebeles of the Hetosa district were considered as a study population.

#### 2.3.3 The study population for qualitative data

All postnatal mothers and all traditional birth attendants (TBAs), all health extension workers (HEWs), and all grandmothers in selected kebeles were considered as a study population.

### 2.4 Eligibility Criteria

#### 2.4.1 Inclusion criteria for quantitative data

All mothers who gave birth in the last 6-month period preceding the study were included in the study.

#### 2.4.2 Exclusion criteria quantitative data

All mothers who stay in the study area for less than 6 months.

All mothers who gave birth to died fetuses or stillbirths.

All mothers whose newborns stay in NICU more than three days after delivery.

#### 2.4.3 Exclusion criteria for the qualitative part

Participants interviewed in the quantitative survey were excluded from in-depth interviews.

### 2.5 Sample size determination

#### 2.5.1 Sample size determination for quantitative study

The sample size for this particular study was determined by using a single population proportion formula using a basic assumption of a 95 % confidence level and, a 5 % margin of error by considering the proportion of safe cord care was 68.3 % with a study conducted in Nekemte city Oromia region, Ethiopia [17].

$$n_{i}=\frac{z^{2}P\left(1-P\right)}{d^{2}},\frac{\left(1.96\right)2\;0.683\;\left(\;1-0.683\right)}{\left(0.05\right)2}=333$$

Where,

Z = standard normal distribution value = 1.96 at

α value of 0.05.

W = margin of error = 0.05.

Using Design effect = 1.5.

Since it is a multi-stage sampling technique by using the design effect and multiplying by 1.5 becomes 500 and Contingency (for non-response = 10 %) = 50 the final sample size is n_f_ = 550.

##### 2.5.1.1 Assumptions

Ni = initial sample size; 333;

NF=Final sample size = 550;

Confidence level which will be 95 %;

P = proportion = 68.3 %;

d = the margin of error will be taken as 5 %.

#### 2.5.2 Sample size determination for qualitative

Qualitative data were collected using a total of twelve In-depth interviews which involved postnatal mothers and from Key Informants. Key informant interviews were conducted among Traditional Birth Attendants (TBAs), grandmothers, and Health Extension Workers (HEWs) in the community until information saturation was obtained.

### 2.6 Sampling procedure

#### 2.6.1 Sampling procedure for quantitative data

**Fig. 1:**
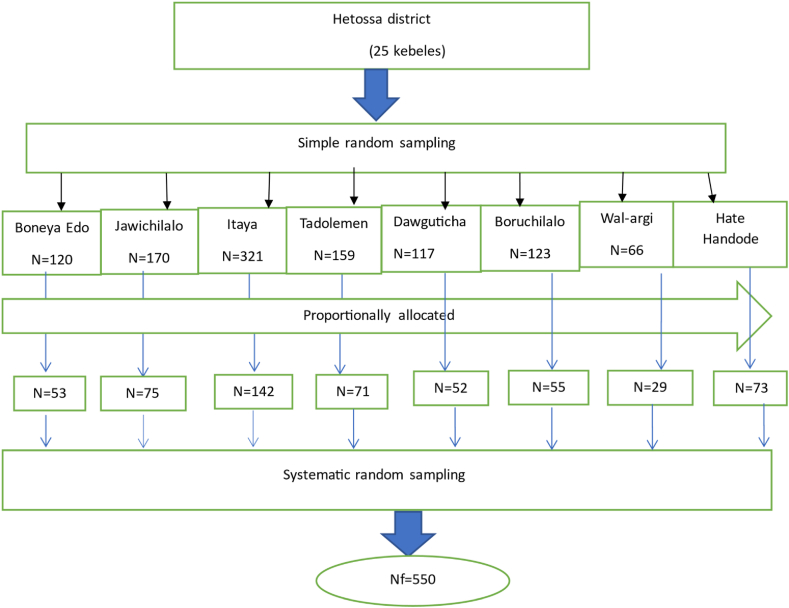
schematic representation of sampling technique to assess cord care practices among mothers who gave birth in the last six months in Hetosa district, Arsi zone, Ethiopia, 2021.

Multi-stage sampling technique was used to select the study participants. The study was conducted in eight Kebele's of hetosa district which is 30 % of the total kebeles. From twenty-five Kebele's found in the district, eight (seven rural and one urban) Kebele's were selected using simple random sampling method. The sample size was allocated to each Kebele proportional to the number of women who gave birth in the last six months from selected Kebele. Skip interval was determined for each kebele by dividing the estimated population by the respective sample size (i.e. Kth = N/n). The expected total number of women who gave birth in the last six months in selected kebeles was N = 1241. Hence, each participant was selected by systematic random sampling with a skip interval of 2 at each kebele. Using the lottery method, 1 was selected and used as a starting number based on the mother's registration book. Subsequently, every other mother was included until the desired sample size was achieved (Fig. 1).

$$K=\frac{N}{n_{f}}$$

$$K=\frac{1241}{550}=2.25\approx 2$$

Where, N = total Number of mothers who gave birth in the last six months in selected kebeles.

n_f_ = final sample size;

K=Sampling interval.

#### 2.6.2 Sampling procedure for qualitative data

The purposive sampling technique was used for the qualitative study. The open-ended questionnaire was prepared to obtain an in-depth understanding of neonatal cord care practices and associated factors. A total of 12 in-depth interviews (IDIs) were conducted. The IDIs involved 4 mothers, 3 traditional birth attendants (TBAs), 3 Health Extension Workers (HEWs), and 2 grandmothers all of whom were selected purposively based on their close relation with mothers and neonates, involvement in the community and an assumption to be the richness of information on the topic of the study. The number of IDIs was determined based on the level of saturation of the required information.

### 2.7 Variables of the study

#### 2.7.1 Dependent variables

❖ Umbilical cord care practice

#### 2.7.2 Independent variables

❖ Socio-demographic variables: Age of respondents, Marital status, Residence, Educational status, Religion, Ethnicity, Monthly income, and Occupation
❖ Obstetrical and maternal health service variables: Antenatal care, Place of antenatal care, Number of pregnancies, Number of live births, Number of live children, Place of birth, Postnatal checkup, Birth attendance, and Sex of child.
❖ Cultural and traditional variables: substances application on cord, Type of substance applied, Frequency of application, Time of application, Source of advice about substance applied
❖ Mothers' Knowledge of umbilical cord care practice and source of information about cord care practice.

### 2.8 Operational definitions and terms of definition

Umbilical cord care practice: All types of care of the umbilical cord are administered by a mother postpartum, including topical applications of various substances to the umbilical cord stump from birth to when it naturally falls off [18].

**Dry cord care**: Refers to care of the cord whereby no chemical substance is applied to the cord, only cleaning with plain water and soap until it falls off [19].

**Good cord care:** Defined as keeping the cord, clean and dry without application of any substance on the cord stump except medically indicated medications like chlorhexidine after cutting the umbilical cord [20].

**Poor cord care:** was defined as when the substance was applied to the cord stump of a newborn other than Chlorhexidine after cutting the umbilical cord.

**Good knowledge of mothers:** Knowledge was "good" for mothers who correctly responded to 3 or more knowledge related questions [21].

**Poor knowledge of mothers:** Knowledge was "poor" for mothers who correctly responded to less than 3 knowledge related questions correctly.

### 2.9 Data collection tool and procedure

#### 2.9.1 Data collection tool and procedure for quantitative data

A pre-tested, structured questionnaire was used to collect quantitative data. The instrument was derived from standard data collection tools and related literature. It was pre-tested for consistency of responses by taking 5 % of the sample size, conducted in a similar population but out of the selected kebeles. Necessary modifications were made accordingly before the actual data collection. To maintain validity, the questionnaire was prepared in English then translated into Afan Oromo and Amharic versions and back-translated to English by the principal investigator and language experts to ensure consistency and accuracy. The data were collected by trained 8 BSC holders with health-related fields from selected Keble's. Two supervisors were assigned to facilitate data collection. During data collection, the participants were told the significance of the research, and the consent of the respondents was taken.

#### 2.9.2 Data collection tool and procedure for qualitative data

To generate qualitative information, an in-depth interview (IDI) was performed/conducted by the principal investigator. Each interview was audio recorded and taken after obtaining oral voluntary consent from the interview participants.

### 2.10 Data quality control

#### 2.10.1 Data quality control for quantitative data

The two-day training was given to all data collectors on instructions on how to fill out the questionnaire and supervisors to facilitate data collection. The principal investigator& supervisors were closely following the day-to-day data collection process and ensure completeness and consistency. All the completed questionnaire was returned daily. Data were checked for completeness, accuracy, clarity, and consistency before being entered into the software. Proper coding and categorization of data were maintained for the quality of the data to be analyzed.

#### 2.10.2 Data quality assurance for qualitative data

Open-ended IDI questions taken from the literature were used for qualitative data collection [19] The data were collected by the principal investigator by audio recording and note-taking. Trustworthiness or quality of the data analysis was ensured by data source triangulation by interviewing different bodies in the community. Furthermore, allocating sufficient time; to collect data and maintaining an objective and impartial view further added to the reliability of the data.

### 2.11 Data Processing and analysis

#### 2.11.1 Data Processing and analysis for quantitative data

The pre-coded data were entered into Epi data version 3.1 software and then it was exported to SPSS version 25 for statistical analysis. A score of one was given for good cord care practice and zero for poor cord care practice. A binary logistic regression was used to identify the association of the independent variables with the dependent variable. Each variable that has p-value less than 0.25 was added to the final model to control the confounders. Variables that have a p-value <0.05 with a 95 % confidence interval in the final model were declared statistically significant. Knowledge of mothers on newborn cord care was assessed using a series of six close-ended questions and a composite variable was generated from these questions to categorize mothers as having “Good/poor” knowledge. A scoring system was used to analyze responses to closed-ended questions on Knowledge: 1 = coded as correct response and 0 = coded as incorrect response. Accordingly, mothers who correctly responded to at least 3 questions were categorized as having a good knowledge of newborn cord care. The descriptive data were presented using frequency, tables, figures, mean, and standard deviation. Hosmer and Lameshow goodness of fit test was conducted to test the model fitness and the model was adequate (p = 0.266). Multicollinearity was checked by using VIF and it was <10.

#### 2.11.2 Data Processing and analysis for qualitative data

Primarily, audio-recorded data were heard repeatedly until the principal investigator became intimately familiar with the contents. The audio-recorded qualitative data were transcribed into the English language. Then, codes or terms were identified and tallied to come up with some categories, which were later used to establish themes based on the objective of the study. Unique concepts were identified and trials were made to elaborate more and report on the final result. Finally, thematic analysis was done manually and the findings were triangulated with the quantitative one.

Ethical approval was obtained from the Institutional Ethical Review Committee (IERC) of Debre Markos university (DMU), college of health science. Following the approval by IERC, an official letter of cooperation was written to the Hetosa Woreda health administration office, and in turn, the district health administration office wrote letters to each selected kebeles administration office to get permission and cooperation. The written informed consent from the respondent was obtained. The confidentiality and privacy of the respondents was assured. Participants were given the chance to ask any doubt about the study and made free to refuse or stop the interview at any moment they wanted.

## 3 Result

### 3.1 Quantitative result

#### 3.1.1 Socio-demographic characteristics

A total of 541 mothers who gave birth in the last six months participated in this study giving a response rate of 98.36 %. The mean (mean ± SD) age of the respondents was 28.41 ± 5.72 years. Of all participants, 488 (90.2 %) mothers were married and 471 (87.1 %) were Oromo in ethnicity. Among the total participants, 263 (48.6 %) mothers attended primary school and about 286(52.9 %) women were orthodox in religion. About occupational status housewife 397 (73.4 %) take a larger proportion. The majority 405(74.9 %) mothers were rural residents and 224 (53.5 %) respondents used radio as a mass media source of information (Table 1).

**Table 1:** Socio-demographic characteristics of mothers who gave birth in the last six months in Arsi zone, Hetosa district, Ethiopia, 2021, (n = 541).

Variable	Category	Freq	Variable	Category	Freq
Age	≤24 years	135(25.0 %)	Maternal occupation	Housewife Farmer Government employer Other(merchant, daily labourer, student)	397(73.4 %) 21(3.9 %) 69(10 %)
	25–32 years	281(51.9 %)			
	≥33years	125(23.1 %)			
Marital status	Single	26(4.8 %)	Do you have a source of Information/mass media	Yes No	419(22.6 %) 122(77.4 %)
	Married	469(86.7 %)			
	Other (windowed, divorced, separated)	46(8.5 %)			
Ethnicity	Oromo	471(87.0 %)	Type of information source you have	Radio Television	224(53.5 %) 195(46.5 %)
	Amhara	42(7.8 %)			
	Other(Tigree, Gurage, Woliyta)	28(5.2 %)			
Religion	Orthodox	285(52.7 %)	Monthly income	≤1999 2000–3999 ≥4000	282(52.1 %) 154(28.5 %) 105(19.4 %)
	Muslim	189(34.9 %)			
	Protestant	56(10.4 %)			
	Other (catholic)	11(2.0 %)			
Education level	No formal education	81(15.0 %)	Residence	Rural Urban	405(74.9 %) 136(25.1 %)
	primary	263(48.6 %)			
	secondary and above	197(36.4 %)			

##### 3.1.1.1 Obstetric and maternal service factors

Attendance of ANC was high among the respondents with 505 (93.3 %) reporting to have attended at least one ANC visit. Most 255(50.5 %) respondents had made four or more ANC visits in their most recent delivery.

From the total mothers who participated in this study 320(59.1 %) were multigravidas. The majority of the respondents delivered their babies at a health facility 418 (77.3 %) while 123 (22.7 %) had home deliveries. Almost all of the respondents delivered their babies at health facilities attended by health professionals. On the other hand majority of those who had their birth in the home were attended by families 46(37.7). Among the total participants, 216 (39.9 %) mothers had postnatal care after their deliveries of recent baby. Slightly more than half of the respondents 295(54.5) gave male babies (Table 2)

**Table 2:** Obstetric characteristics of mothers who gave birth in the last six months in Arsi zone, Hetosa district, Ethiopia, 2021, (n = 541).

Variable	Category	Frequency	Percent %
Number of ANC visit	One	23	4.6
	Two	80	15.8
	Three	147	29.1
	≥four	255	50.5
Place of ANC	Private clinic	72	14.3
	Hospital	58	11.5
	Health center	284	56.2
	Other (health post)	91	18.0
Gravidity	Primigravida	142	26.2
	Multigravida	320	59.2
	Grand multipara	79	14.6
Place of birth	Home	123	22.7
	Health facility	418	77.3
Birth attendant	Health professional	418	77.3
	TBA	45	8.3
	Family	46	8.5
	Friend	8	1.5
	Other	24	4.4
Baby's sex	Male	295	54.5
	Female	246	45.5

ANC = antenatal care, TBA = traditional birth attendant.

#### 3.1.2 Source of information and knowledge of mothers on cord care

Slightly more than half 298(55.1 %) respondents received information regarding umbilical cord care and most of them 217(72.8 %) received the information from health professionals either before or after the delivery of their recent baby. Among the participants who had ANC visits only 82 (16.2 %) said they were informed about umbilical cord care during their ANC visit (Table 3)

**Table 3:** Source of information of mothers who gave birth in the last six months in Arsi zone, Hetosa district, Ethiopia, 2021, (n = 541).

Variable	Category	Frequency	Percent %
Received information regarding UCC before or after delivery	Yes	298	55.1
	No	243	44.9
A person who gave the information regarding UCC	Health professional	217	72.8
	Family	31	10.4
	Neighbor	28	9.5
	TBA	12	4.0
	Mass media	7	2.3
	Other (friend)	3	1.0
A place where the information received regarding UCC	Health facility	194	65.1
	Home	67	22.5
	Neighbor	30	10.1
	Other	7	2.3
Umbilical cord care education during ANC	Yes	82	16.2
	No	423	83.8

ANC = antenatal care, UCC = umbilical cord care.

With regard to knowledge, 91.9 % of the respondents acknowledged that they always wash their hands with soap and water before umbilical cord care. However, 283 (52.3 %) of them knew that no substance was applied to the cord stump except chlorohexidine. Of the participants, 450(83.2 %) and 396(73.2 %) knew that cord-cutting and cord-tying material should be sterile respectively (Table 4).

**Table 4:** Knowledge of mothers who gave birth in the last six months in Arsi zone, Hetosa district, Ethiopia, 2021, (n = 541).

Variables	Category	Frequency	Percent(%)
Hand wash with soap and water before UCC	Yes	497	91.9
	No	44	8.1
No substance should be applied on the cord stump except chlorohexidine	Yes	283	52.3
	No	258	47.7
The diaper should not cover the cord	Yes	185	34.2
	No	356	65.8
Newborn bathing should be sponge bath till the cord is cut off	Yes	152	28.1
	No	389	71.9
Cord-cutting material should be sterile	Yes	450	83.2
	No	91	16.8
Cord tying material should be sterile	Yes	396	73.2
	No	145	26.8
Overall knowledge	Good	380	70.2
	Poor	161	29.8

UCC = umbilical cord care.

**Fig. 2:**
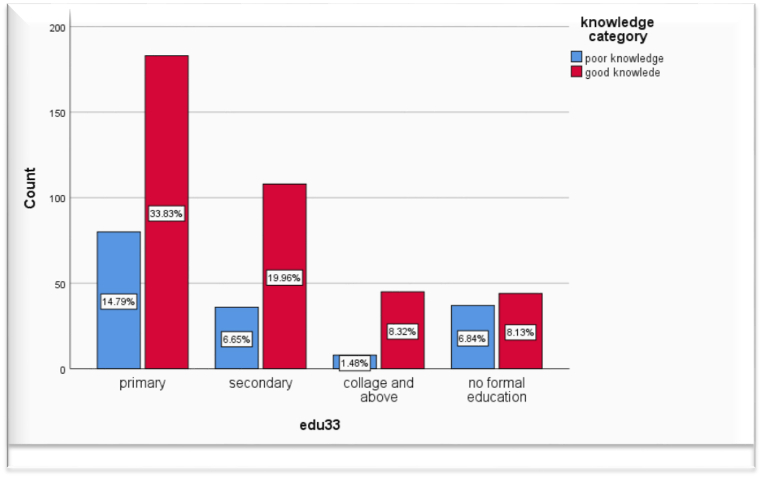
Knowledge level about umbilical cord care among mothers who gave birth in the last six months in Arsi zone, Hetosa district, Ethiopia, 2021, (n = 541).

Regarding knowledge of handling umbilical cords around 70.2 % of study participants had good knowledge and 29.8 % had poor knowledge (Fig. 2)

#### 3.1.3 Umbilical cord care practices among mothers

Overall good cord care practice was found to be 53.4 % [95%CI, 49–58]

Of the respondents practiced good cord care, 217(40.1 %) did provide dry cord care and respondents, 324 (59.9 %) applied substances on the cord.

**Fig. 3:**
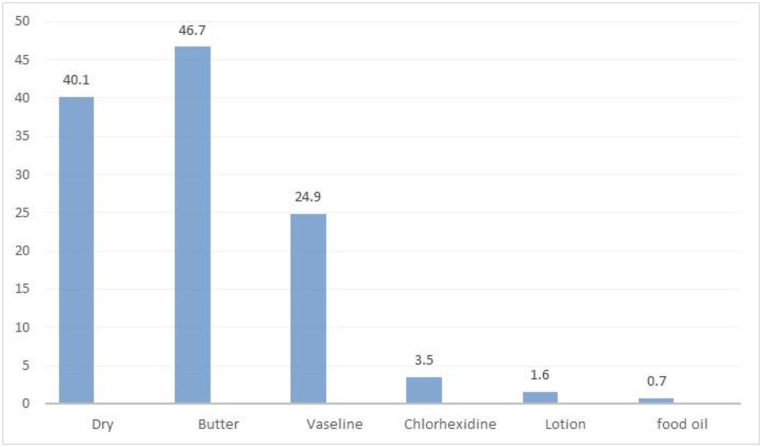
Common substances applied to the cord stump among mothers who gave birth in the last six months in Arsi zone, Hetosa district, Ethiopia, 2021, (n = 541).

Most study participants around 217(40.1 % of them applied dry cord care, 206(46.7 %) of them applied butter,110(24.9 %) of participants applied Vaseline (petroleum jelly), 7(1.6 %) lotion, 3(0.7 %) food oil, 1(0.2 %) alcohol and 5(1.1 %) of them apply applied commercially available substance such as TTC. But 109(24.7 %) of the respondents applied Chlorhexidine as single or in combination with other substances. However, only 72(13.3 %) applied Chlorhexidine alone appropriately (once daily for seven days) (Fig. 3).

Almost all participants applied those substances for different reasons. Among them, 123(22.7 %) applied substances to moisturize and prevent dryness of the cord stump whereas, 116(21.4 %) applied to facilitate cord separation. Others 84(15.5 %) applied ∗that substance to promote of the respondents who applied the substance, 129(23.8 %) mothers started to apply the substance on the second day, and 80(24.7 %) applied once daily. Regarding a person applying a substance on the cord, in 189(58.3 %) cases the substance was applied by the baby's mother and of those respondents who applied substance 210(64.8 %) always washed their hands before they applied the substance. Among those respondents who applied substance on the cord stump majority of them 222(68.5 %) had received information about the substance they applied and most of them 133(59.9 %) received the information from family members.

Various instruments were used to cut the cord. Common instruments used to cut the cord among mothers who deliver at health facilities were surgical blades and boiled/new scissors. However, among participants delivered at a health facility, 180(42.9 %) respondents didn't remember the material used to cut the cord. But 110(99.1 %) mothers who deliver at home use new razor blades. However, 13(2.4 %) used old scissors/old razor blades in case of home delivery. Regarding materials used to tie the cord, a cord clamp was used to tie the cord in half of the respondents 274(50.6 %), otherwise, 105(19.4 %) participants didn't remember the material used to tie the cord. About 18(14.6 %) mothers who deliver at home didn't use anything to tie the cord or leave the cord without tying.

Among study participants, the majority of mothers wash their child's body by immersion of whole body in water. Out of those interviewed 195 (36.0 %) correctly stated that the cord should be dry after washing their baby's body and majority of them dry their baby by loosely covered using a clean piece of cloth. Of the participants in this study, 480(88.7 %) didn't notice any complication on the cord. However, some of the mothers 61(11.3 %) notice complications such as reddening of the umbilicus 28(5.2 %), pus at the umbilicus 20(3.7 %), cord bleeding 10(1.8 %), swelling of the umbilicus 3(0.6 %). Most of the mothers 444(82.1 %) did allow the cord to separate on its own. But some of them 97(17.9 %) interfere with the cord separation process or try to separate the cord rather than allow the cord to separate on its own.

#### 3.1.4 Qualitative results

For the qualitative component study, four postnatal mothers, three traditional birth attendants (TBA), three health extension workers (HEW), and two grandmothers’ participants were involved in a total of twelve in-depth interviews. The age of the participants ranged from 22 to 70 years with a mean age of 46 years. Participants' educational levels varied from no formal education to college. Regarding their occupation all mothers involved in this study were housewives, two of TBA and all grandmothers were farmers and one TBA was a merchant in their profession. Otherwise, all health extension workers were government employers. The result of this finding was used as a supplement for quantitative study by exploring common cultural cord care practices and factors associated with their practice.

#### 3.1.5 Umbilical cord tying and cutting

The majority of the interviewees in the qualitative findings stated that the cord was cut with a new blade and tied by thread or string if delivery occurred at home. Some of them reported that the boiled new razor blade was used instead if delivery was not emergent. Most of them believed that old blades or scissors should not be used to cut the cord, instead, it is better to buy the new one and keep it as the pregnancy reaches to term. However, there is variability in what is used to tie the cord such as sewing thread and strips of Cloth” netelacherk” in the local language and some of them believe that it is not necessary to tie the cord. Most of the time the cord was cut at a length of one or two joints of a finger but there is variation in cord tying practice.

*“ …. while I attend labor, I always used a new razor blade that I always captured in my pocket which I always boiled before use and I never used razor blade that was used before and I tied the cord with sewing thread or strip of cloth netelacherk in absence of it”* (IDI with A 55 years old TBA).

*“I always measure two joints of fingers to the newborn side and cut to prevent bleeding. It never bleeds if I leave two joints of my finger to the newborn side and I never tie the cord. But the reason behind to the cord bleeding comes from to short cord i.e. if the cord cut less than two finger joints”.* (IDI with 70 years old TBA)

*“I have given nine children at home and I never faced any complication until my recent delivery in which all attendants measured the cord with finger joints and cut with a new razor blade that I bought from the market or a knife-like blade given from health post and the cord to the newborn side was never tied”* (IDI with 41 years old mother).

#### 3.1.6 Common substances applied on cord stump

##### 3.1.6.1 Fresh butter

In line with the quantitative finding, most of the participants in the qualitative study believe that the application of fresh butter is mandatory on and around the stump, whether delivery occurs at home or in a health facility.

As reported by the majority of mothers and grandmothers, applying fresh butter to the cord stump immediately after cutting or a few days later will protect the baby from different harm.

*“… during all my deliveries I always applied fresh butter to the cord after I wash my newborn, which it prevents the cord from different hurts such as sticking to the cloth and associated pain with dryness of the cord. It is also used to facilitate its separation in which mostly not exceed no more than three days to fall if fresh better is applied continuously, and then it is better to hit the separated cord to the neck which prevents the newborn from sleep disturbance”* (A 38 years old IDI participant mother).

##### 3.1.6.2 Vaseline/petroleum jelly

According to their opinion, Vaseline can replace fresh butter if it is not available or used instead of using butter stayed more than three days. If butter is not fresh or stays more than three days it may cause soreness or infection to the umbilical cord site.

*“… In the absence of fresh butter commercially available Vaseline which has no any smell can be used instead since butter stays for more days is too bad to apply”* (A 65 years old IDI TBA participant).

##### 3.1.6.3 Alcohol

Others believe that alcohol helps to stop bleeding sometimes caused by inappropriate cord cutting i.e. the cord might bleed heavily if it is too short or less than two joints of a finger. But they never notice that the reason behind cord bleeding is associated with cord tying.

*“In the past, we called the traditional birth attendant (TBA) in our village if the cord didn’t stop bleeding. Thus, once TBA applies alcohol that she took from the health facility, the cord stops bleeding immediately and never bleeds again”* (A 60-year-old IDI participant's grandmother).

Although most mothers weren't aware of as chlorhexidine helps with cord care, all health extension workers who participated in this study believed that nothing should be applied to the cord except medically indicated substances chlorhexidine. Although cord care education during antenatal and postnatal care has a great contribution to preventing harmful cord care practices, the education regarding cord care wasn't given strictly.

In addition, the availability of chlorhexidine helps the health extension worker to educate and remind the mother how to give care to her newborn cord which also helps to replace harmful substances applied at home. But currently, these antiseptics have not reached every health post.

*“I educate mothers not to apply other harmful substances such as cow butter and Vaseline other than chlorhexidine while I distribute this antiseptic, but in the absence of it I forgot to educate them about the care given to the cord at the time of their last antenatal visit”* (IDI with 28 years old HEW).

#### 3.1.7 Reason for substance application

In line with the quantitative study, most of the participants in the qualitative study believed that, if the cord didn't separate within a day, it may cause pain while it dries and sticks to the cloth.

#### 3.1.8 Facilitate cord separation

Most of the participants in the qualitative study believed that, if fresh butter is not applied to the cord, the cord didn't separate within a day or it may take more than a week, which may lead th∗e cord to dry and stick to the cloth.

*“I recommend mothers to apply fresh butter or Vaseline if the cord doesn’t separate within three days, because if the cord doesn’t fall within three days it becomes dry and causes severe pain, otherwise it is not obligatory to apply that substance if the cord falls within three days”* (IDI with 55 years old TBA).

Others believe that the separation of the cord depends on the mother's lifestyle or on what the mother eats or drinks otherwise the cord falls within a few days if the mother is rich in what she eats or drinks.

*“Sometimes mothers apply Vaseline if the cord didn’t fall within three days but this will not happen if the mother is prosperous and drinks milk”* (IDI with 65-year-old grandmother).

*“On the rest of my children, I didn’t apply anything to the cord stump and the cord fell within four to five days but on my recent child, I applied Vaseline and the cord fell within three days as I was told by TBA in our village”* (IDI with 32 years old mother).

Even if health extension workers told mothers not to apply anything to the cord, some mothers especially those who are aged or who have several pregnancies didn't stop to apply those harmful substances. Rather they would like to practice what they have already done before rather than accept education given by health professionals**.**

*“Some mothers said you recommend not to apply anything to the cord, but I applied fresh butter to the cord since I already applied it to the rest of my children and it didn’t hurt any of them or I never saw any complication rather it facilitated it’s separation to fall within few days”* (IDI with 28 years old HEW).

This indicates that the acceptability of mothers from what they receive from health professionals is less than from what they already experience or from what they have already seen or heard from expert persons around them. This indicates that the mothers trust fewer health professionals than those other persons around them.

According to most of the mother's ideas, the substance they apply on the cord was informed by a person assisting by their recent birth especially TBA in case of home delivery.

#### 3.1.9 Prevent the cord not to drying and sticking to the cloth

The other reason for substance application to the cord stump was the fear of the cord drying and associated pain. Most of them believed that, once the cord dries it may cause pain while it dries and sticks to the cloth.

*“If we apply fresh butter or Vaseline the cord never dries and becomes soft otherwise once the cord dries, it will stick to the cloth, which leads to severe pain that the newborn cannot tolerate” (IDI with 22 years mother).*

*“I always prepare fresh butter during all my delivery. If we never apply butter to the cord, the cord becomes as hard as a bone the cord sticks to the cloth, and the baby will cry due to the pain cord sickness of the cloth. Thus, the butter prevents it from dryness”* (IDI with 41-year-old mother).

#### 3.1.10 Factors associated with good cord care practice

In the study, all the preliminary assumptions such as model fitness and collinearity were.

Checked and found it to be satisfactory. Consequently, in bivariable logistic regression, the age of the mother, educational status of the mother, marital status, mother's occupation, having mass media, gravidity, place of birth, and knowledge status of the mother were included in multivariable analysis. However, after controlling the effect of confounding in multivariable analysis the final result confirmed that mothers' age, education, place of birth, and level of knowledge on umbilical cord care were found to be statistically significant at p-value <0.05.

Hence, those who had primary, secondary, and above educational status had 5.26 times (AOR = 5.26, 95 % CI: 2.50, 11.04) and 3.63 times (AOR = 3.63, 95 % CI: 1.61, 8.18) more likely to practice good cord care than mothers who had no formal education, respectively. The odds of having a good cord care practice among age groups of ≤ 24years and 25–32years was 4.56 times (AOR = 4.56, 95 % CI; 2.08, 9.96) and 3.70 times (AOR = 3.70, 95 % CI; 1.98, 6.94) higher than mothers whose age ≥ 33 respectively. Hence younger mothers practice good cords as compared to older ones. The odds of delivering in a health facility was 5.09 times (AOR = 5.09, 95 % CI; 2.95, 8.78) was found to significantly associate good practice of cord care than among those who delivered outside the health facility and mothers who had good knowledge of newborn cord care practice were eight times more likely to practice good cord care than those who had poor knowledge (AOR = 8.58, 95 % CI; 5.09, 14.46) (Table 5)

**Table 5:** Bivariable and multivariable logistic regression model predicting the likelihood of good cord care practice among mothers who gave birth in the last six months in Arsi zone, Hetosa district, Ethiopia, 2021, (n = 541).

Variables	Practice		COR (95%CI)	AOR (95%CI)	P-value
	Good(%)	poor (%)			
**Age group (years)**					
≤24	92(31.8 %)	43(17.1 %)	7.08(4.08, 12.28)	4.56(2.08, 9.96)	0.000^a^ 0.000^a^
25–32	168(58.1 %)	113(44.8 %)	4.92(3.04, 7.94)	3.07(1.98, 6.94)	
≥33	29(23.2 %)	96(38.2 %)	1	1	
**Educational status**					
No formal education	15(5.2 %)	66(26.2 %)	1	1	0.000^a^ 0.002^a^
Primary	148(51.2 %)	115(45.6 %)	5.66(3.07, 10.43)	5.26(2.50, 11.04)	
Secondary and above	126(43.6 %)	71(28.2 %)	7.80(4.15, 14.68)	3.63(1.61, 8.18)	
**Mothers' occupation**					
Housewife	192(66.4 %)	205(81.3 %)	0.33(0.18, 0.58)	0.46(0.20, 1.06)	
Farmer	10(3.5 %)	11(4.4 %)	0.32(0.11, 0.88)	1.03(0.24, 4.33)	
Government employer	36(12.5 %)	18(7.1 %)	0.70(0.32, 1.54)	0.81(0.28, 2.30)	
Other	51(17.6 %)	18(7.1 %)	1	1	
**Marital status**					
Single	20(6.9 %)	6(2.4 %)	1	1	
Married	240(83.1 %)	229(90.9 %)	0.31(0.12, 0.79)	0.59(0.13, 2.23)	
Other	29(10.0 %)	17(6.7 %)	0.51(0.17, 1.52)	1.20(0.26, 5.49)	
**Access to mass media**					
Yes	237(82.0 %)	182(72.2 %)	1.75(1.16, 2.63)	1.58(0.91, 2.73)	
No	52(18.0 %)	70(27.8 %)	1	1	
**Gravidity**					
Primigravida	101(34.9 %)	41(16.3 %)	6.39(3.46, 11.76)	1.41(0.58, 3.41)	
Multigravida	166(57.4 %)	154(61.1 %)	2.79(1.63, 4.78)	0.83(0.39, 1.73)	
Grand multipara	22(7.6 %)	57(22.6 %)	1	1	
**Birthplace**					
Health facility	260(90.0 %)	159(63.1 %)	5.24(3.30, 8.31)	5.09(2.95, 8.78)	0.000^a^
Home	29(10.0 %)	93(36.9 %)	1	1	
**Knowledge level**					
Good	260(90.0 %)	120(47.6 %)	9.86(6.24, 15.56)	8.58(5.09, 14.46)	0.000^a^
Poor	29(10.0 %)	132(52.4 %)	1	1	

a = p-value <0.05, COR = crude odds ratio and AOR = adjusted odds ratio.

## 4 Discussion

Although the majority of women 380(70.2 %) had good knowledge of cord care, only 53.4 % [95%CI, 49%–58 %] of women had good cord care practice. Among 289(53.4 %) of the respondents had good cord care, 217(40.1 %) did provide dry cord care and 72(13.3 %) applied Chlorhexidine appropriately. The finding of this study was almost nearly similar to the study done in Chencha district, southern Ethiopia 52.9 % [22].

But this finding was higher than the study conducted in Hossana town, Hadiya zone, southern Ethiopia, 32.9 %, in Garissa county, Kenya which was 43 %, a population survey in eastern Uganda 38 % and in Cameroon 19.7 % [18,[23], [24], [25]]. And this difference might be due to relatively a recent increase in antenatal care visits and institutional delivery services. The difference can also be a result of chlorhexidine provision among mothers. In addition, the current community-based interventions by health extension workers might have contributed to the reduction of harmful cord care practices among mothers in the study area. This is supplemented by qualitative findings in Pemba Tanzania which revealed that there was consensus among respondents that CHX liquid cord cleansing could be successfully implemented in the community with appropriate education and awareness [26].

Inversely this finding was lower than the study conducted in rural Sidama Zone, Southern Ethiopia 73 %, in Nekemte City, western Ethiopia 68.3 %, in Jimma Zone, Southwest Ethiopia 86.5 % [17,27,28]. The possible explanation for this difference might be due to the presence of socio-cultural differences that exists across the country. The discrepancy can also be a result of differences in residency women that are urban residents will have information that could assist them in making decisions regarding healthy behaviors including maternal and child health education, and promotion including newborn cord care practice.

This study finding was also lower than the studies carried out in Ibadan, Nigeria 61.4 %, 77.8 %, 2013 edition of the Nigeria Demographic and Health Survey (NDHS) 72.2 % [21,29,30]. The reason for this difference might be the socio-cultural difference, geographical area, economic status, and difference in life standard across the countries. The other possible reason also might be due to the difference in the variables included in measuring cord care practice. Some studies included only cord cleaning or the substance used for cord cleaning and others included the substance applied and the materials used to cut and tie the cord. However, in this study, the substance applied after cord cutting was used to measure mothers' cord care practice.

This finding was supplemented by qualitative findings. In this study 59.9 % of mothers responded that substances were applied on the stump; the overall percentage of respondents who applied substances on cord stump is higher than that found in four regions of Ethiopia (Amhara, Oromia, SNNPR, and Tigray) in 21 % and butter is the most commonly used substance in both studies [31].

With regard to the reason for application this study was consistent with a systematic review held in low and middle-income countries in which the desire to promote healing and hasten cord separation are the underlying beliefs related to the application of substances to the

Umbilical cord [12]. With regard to the material used to cut the ∗cord, this study was similar to the study conducted in Tanzania in which all TBA used new razor blades to cut the cord [26]. Some of the mothers and TBA never tied the cord in case of home delivery. This finding was consistent with the study conducted in Southern Ethiopia, rural Sidama zone in which 11.6 % of cases never tied the cord [27].

In the current study, educated mothers were more likely to practice good cord care than those who had no formal education. A formally educated mother is more exposed and informed than an uneducated and as such can make better decisions regarding her child's health and will likely possess a much better health-seeking behavior. This study is supported by study findings conducted in Benin City, Edo State, Nigeria, and Calabar Metropolis Cross River State, Nigeria [5,32].

This finding is also in keeping with studies that show that the higher the level of maternal education, the better the health-seeking behavior and thus exposure to a better knowledge of childcare practices. This study is supported by study findings conducted in the Volta region of Ghana, in Abakaliki, Ebonyi State, South-East Nigeria, and in Yenagoa Local Government Area, Bayelsa State, Nigeria [1,2,33].

In this study, younger women were more likely to practice good cord care than those who are older women. The possible reason behind this can be those younger mothers were more likely educated than older ones. In addition, older mothers may not be equipped with the latest cord care guidelines which have been modified in recent times. Another explanation may be as the age of women increases the probability of having other previous pregnancies or children increases. This may lead women to apply more cultural practices received from their experience. This finding is opposed to studies conducted in Ibadan, Nigeria, in Benin City, Edo State, Nigeria, and the 2013 edition of the Nigeria Demographic and Health Survey (NDHS) which show that older women practice better cord care than younger mothers [5,29,30]. This difference may be a result of the fact that older mothers tend to have delivered more times thann younger mothers and as such are more experienced in child care as they may have learned hard lessons as well following harmful cord care over the years.

This study finding also supported the qualitative study that older mothers are more frequently like to apply some non-recommended substances which is harmful to the newborn. This finding is consistent with a study conducted in the voltage region of Ghana [33]. This might be associated with mothers' less trust in health workers or due to their past influence from what they see, hear, or exercise. According to the interview with health extension workers, aged mothers refused to accept the recommended cord care practices.

According to this study finding, mothers who deliver in the health facility were more likely to practice good cord care than those who deliver outside the health facility. The possible reason for this is that as the possibility of the mother delivering in a health facility increases, the opportunity for this mother to aware more of her newborn care increases. This is due to Facility childbirth had a strong association with appropriate newborn care probably because of the ease with which medical advice on appropriate newborn practices including cord care can be obtained and given [29].

This finding is supported by study findings conducted in two communities of Plateau State, North Central Nigeria, in Yenagoa Local Government Area, Bayelsa State, Nigeria, and rural contemporary Nigeria setting [2,21,34] which show that mothers who deliver in recognized health facilities are more likely to practice better cord care.

According to this study finding, mothers who had overall good knowledge were more likely to practice good cord care than mothers who had poor overall knowledge. The possible explanation for this is that once the mother knows the recommended practices on the newborn umbilical cord, the possibility of practicing what she already knows is higher than what she didn't know. This study finding was supported by the study employed in Chencha District, Southern Ethiopia, and Hossana town, Hadiya zone, southern Ethiopia [22].

However this finding is inconsistent with studies conducted in Ibadan, Nigerians, in which knowledge about cord care practice has no significant change in their practice [29]. The inconsistency can be due to, Mothers who are aware of the effects of harmful cord care practices are less readily going to practice it. It can also be associated with a low level of mothers' trust in what they receive from health professionals than what they know or hear. This might be due to the influence of culture around them. The most common influencers in the community can be families such as mothers and grandmothers or traditional birth attendants most of the time they have a great acceptability to the community.

## 5 Conclusion

This study indicated that the magnitude of newborn cord care practice was low among mothers who gave birth in the last six months in the district.

Age, educational status, place of delivery, and knowledge level regarding umbilical cord care were statistically significant predictors of good cord care practice.

Therefore, age, education, health facility delivery, and having good knowledge of umbilical cord care expose the mother to a good cord care practice.

### 5.1 Recommendation

#### 5.1.1 Health care professionals

Provide health education regarding newborn cord care during at all levels of contact with the mother with increased reminders during the antenatal period.

Good health-seeking behavior and delivery at health facilities to effectively promote good cord care practice should be encouraged.

Update health care providers' skills and knowledge or in-service re-education on appropriate umbilical cord care.

Should encourage mothers to apply chlorhexidine appropriately if available otherwise encourage dry cord care.

#### 5.1.2 Health facility

Facilities should have a standard protocol for umbilical cord care which should be communicated to all women and careers by their healthcare providers.

#### 5.1.3 District health bureau

Strengthen health extension workers to counsel and advise the mothers about cord care practice.

Arrange different community-based interventional strategies such as community-based education to address the problem.

Increase the availability of chlorhexidine which replaces the application of other harmful substances.

#### 5.1.4 Healthcare managers and policymakers

Strengthen policies and strategies focused on women's education.

Use this study as an input to carry out further investigation and make the problem addressed through different strategies.

#### 5.1.5 Educators

Use the findings in teaching-learning process.

#### 5.1.6 Researchers

More studies, analytical and interventional should be done to obtain the determinants of harmful cord practices and to make further recommendations to decrease the prevalence of harmful cord care.

A study on assessing cord care practices at health facilities is needed to identify the care provided by health care providers.

A similar or comparative study needs to be done in the country for ease of generalization.

## 6 Strength and limitation of the study

### 6.1 Strength

Since the study conducted use of community-based study and inclusion of rural kebeles which increase the generalizability of the study is the strength of this study. It also tries to address the cultural cord care practice of mothers with qualitative findings in the community.

### 6.2 Limitation

The cross-sectional nature of the study makes it impossible to establish a temporal relationship between newborn cord care practices and identified risk factors.

## CRediT authorship contribution statement

**Genat Balcha Abdi:** Writing – review & editing, Writing – original draft, Visualization, Software, Methodology, Investigation, Formal analysis, Data curation, Conceptualization. **Bekalu Kassie Alemu:** Writing – review & editing, Writing – original draft, Visualization, Validation, Supervision, Software, Resources, Methodology, Investigation, Formal analysis, Data curation, Conceptualization. **Tensae KassaYizengaw:** Writing – review & editing, Writing – original draft, Visualization, Validation, Supervision, Software, Methodology, Investigation, Formal analysis, Data curation, Conceptualization. **Beker Ahmed Hussein:** Writing – review & editing, Writing – original draft, Validation, Data curation.

## Data availability statement

**D**ata supporting the findings of this study are available in Figshare at URL (10.6084/m9.figshare.27924774). Additional data and materials related to this study are available from corresponding author upon reasonable request (Beker Ahmed, bekinan2023@gmail.com).

## Ethical approval

This study was approved by Institutional Research Ethical Review Committee (IRERC) of Debre Markos University, college of health science with reference number HSC/R/C/ser/co/318/11/13, dated April 01, 2021 with relevant guidelines and regulations Following the approval by IRERC, an official letter of cooperation was written to the Hetosa Woreda health administration office, and in turn, the district health administration office wrote letters to each selected kebeles administration office to get permission and cooperation. Written informed consent was obtained from all participants prior to data collection. The confidentiality and privacy of the respondents was assured. Participants were given the chance to ask any doubt about the study and made free to refuse or stop the interview at any moment they wanted and no study participants below the age of 18 years old.

## Funding

A total fund of 2500 Ethiopian birr with a project code of HSC/R/C/ser/co/318/11/13, dated April 01, 2021 was received from the 10.13039/501100021567Debre Markos University Office of Research and Publication directorate director. The funding agency was not involved in the study's conception and design, data collection, participant enrollment, statistical analysis, data interpretation, or manuscript development.

## Declaration of competing interest

The authors declare that they have no known competing financial interests or personal relationships that could have appeared to influence the work reported in this paper.

## Acronyms and abbreviations፡

**ANC**: Antenatal care
**AOR**: Adjusted Odds Ratio
**CHX**: Chlorhexidine
**COR**: Crude Odds Ratio
**EDHS**: Ethiopian Demographic Health Survey
**HEW**: Health Extension Worker
**IDI**: In-Depth Interview
**KAP**: Knowledge, Attitude, and Practice
**NDHS**: Nigerian Demographic Health Survey
**NMR**: Neonatal Mortality Rate
**UCC**: Umbilical cord care
**WHO**: World Health Organization
